# Hadoop Oriented Smart Cities Architecture

**DOI:** 10.3390/s18041181

**Published:** 2018-04-12

**Authors:** Vlad Diaconita, Ana-Ramona Bologa, Razvan Bologa

**Affiliations:** Department of Computer Science and Cybernetics, Bucharest University of Economic Studies, Bucharest 010374, Romania; ramona.bologa@ie.ase.ro (A.-R.B.); razvanbologa@ase.ro (R.B.)

**Keywords:** smart cities, sensors, Hadoop, Spark, Elasticsearch, cloud computing, IoT

## Abstract

A smart city implies a consistent use of technology for the benefit of the community. As the city develops over time, components and subsystems such as smart grids, smart water management, smart traffic and transportation systems, smart waste management systems, smart security systems, or e-governance are added. These components ingest and generate a multitude of structured, semi-structured or unstructured data that may be processed using a variety of algorithms in batches, micro batches or in real-time. The ICT architecture must be able to handle the increased storage and processing needs. When vertical scaling is no longer a viable solution, Hadoop can offer efficient linear horizontal scaling, solving storage, processing, and data analyses problems in many ways. This enables architects and developers to choose a stack according to their needs and skill-levels. In this paper, we propose a Hadoop-based architectural stack that can provide the ICT backbone for efficiently managing a smart city. On the one hand, Hadoop, together with Spark and the plethora of NoSQL databases and accompanying Apache projects, is a mature ecosystem. This is one of the reasons why it is an attractive option for a Smart City architecture. On the other hand, it is also very dynamic; things can change very quickly, and many new frameworks, products and options continue to emerge as others decline. To construct an optimized, modern architecture, we discuss and compare various products and engines based on a process that takes into consideration how the products perform and scale, as well as the reusability of the code, innovations, features, and support and interest in online communities.

## 1. Introduction

We are entering an era where city issues are now problems faced by the entire world. The United Nations estimates that urban population will exceed rural in emerging economies in 2020, and that about 70% of the world’s population will live in cities by 2050 [1]. Some cities are facing a low birth rate, an ageing population, with shrinking budgets, rising unemployment, increasingly inadequate housing and rising crime rates; therefore, the development of smart cities is on the agenda of many governments from all around the world. As the urban population share increases, cities feel urban problems more acutely. Examples include expanding suburbs, air pollution, difficulty in obtaining drinking water, wastewater treatment and sanitation, energy supply, traffic congestion, and waste disposal.

A smart city is a city that uses ICT infrastructure in a flexible, reliable, scalable, affordable, secure and safe way, in order to improve the quality of life of its citizens. It can provide stable economic growth through higher standards of living and job opportunities, welfare, and access to better education [2,3]. Such an innovation can establish a responsible approach towards the environment, which meets the needs of today’s generations, without sacrificing those of future generations [4]. It is able to streamline physical infrastructure-based services, such as transportation (mobility), water, utilities (electric, gas), telecommunications, and manufacturing sectors. A geographic information system (GIS) is essential, as it allows a smart city to treat information taking into account the spatial, geographic localization. GIS systems are used in fields such as land registry (cadastre real estate, water supply networks, telephone networks), urbanism, and territorial and local government (determining location optimal new objectives, population register, organizing the collection of waste and landfilling), geology (inventory and deposits supervision), environment, transport (transport routes optimization), agriculture, environmental protection (analysis performed for various pollutants). Smart buildings and smart homes are an essential part of smart cities, offering residents different facilities, ranging from generating a portion of the electricity to remote controlling, monitoring and auto-adjusting different appliances. In 2011 in Europe, the boom in solar energy installations reached its peak. This was mainly based on policies for supporting renewable energies plus increased awareness of environmental issues. 

Hadoop is the most popular Big Data ecosystem. Starting with the introduction of Yet Another Resource Negotiator (YARN) [5], it is no longer only about batch-oriented processing with MapReduce of data stored in the Hadoop Distributed File System (HDFS). Many Hadoop components and products that can run on Hadoop have emerged, offering support for different programming languages, e.g., Spark supports Python, Java and Scala, and providing easy access to a myriad of machine learning algorithms needed for running an efficient smart city. The main advantage of the Hadoop architecture stack is that it is open-source, so has no license cost. The main disadvantage is its limited support and the notorious security problems of most NoSQL databases and Hadoop ecosystem products. Solutions to offset these problems exist, e.g., client to node encryption, the use of external products like Kerberos, etc. However, implementing these and the rest of the architecture requires highly-qualified personnel.

Even for smart cities, especially in eGovernmence, there is the danger of falling into the “Big Data Hubris”, the impression that having large quantities of data and apparently robust algorithms mean that the more traditional ways of ensuring data quality, determining valid and trustworthy dependencies among data, can be overlooked [6,7]. From the citizens’ point-of-view, there are privacy concerns, the fear that smart cities could be turned into “Big Brothers” that constantly monitor and evaluate their every move. Even more, there is some concern regarding collaborative filtering algorithms, like the Netflix prize winner alternating least squares (ALS) algorithm [8], that have been traditionally used to yield movies or product recommendations by companies like Netflix or Amazon, could be unfairly used by banks or even by local governments to adjust credit scores or citizen scores, for example. The problem with Big Data algorithms is that they can take into consideration not only the actions of the ranked person but also other persons’ actions. Banks could cut someone’s credit card limit because he or she shops in establishments where people who previously shopped had difficulties paying their instalments [9]. While bias and unfairness risks exist when using an “all data is credit data” approach [10], some authors argue that Big Data methods can be useful to evaluate people with limited or no credit history, i.e. that otherwise would not have access to such bank services [11,12]. The unfairness concerns should be carefully and transparently addressed by companies, local governments, or mayoral offices. If transparently used, ALS type algorithms could benefit the community. For example, they could better promote tourist points of interest to different target groups, or to better customize social programs according to recipients.

Using IoT and Big Data analytics for smart tourism and sustainable cultural heritage is discussed in [13], together with the concept of smart and connected communities (SCC). To support an SCC (case study: TreSight, Torino, Italy), a conceptual architecture and a backend architecture that contains a NoSQL database (MongoDB) and an HFS Cluster (XIFI nodes) is presented. 

The combination of smart things and smart spaces, interconnected in smart cities, works because of the Internet of Things (IoT) technology: smartphones, RFIDs, devices, sensors, vehicles, home appliances and network capacity to transmit large volumes of data. The data collected from these providers consists of the knowledge accumulated on the basis of data analysis using classical statistical algorithms, data mining, or machine learning, and can help give value.

So, in order to fully benefit from the massive amounts of data produced by these smart systems, a data management and processing system is needed. The ways in which cloud computing responds to these needs for data collection, storage and management was discussed by Ahuja et al. [14]. The availability of abundant data sources from intelligent devices and smart homes, the rapid progress made in IoT, and Big data technology, make it possible to apply appropriate algorithms to enable intelligent decisions in driving smart city activities. What is noticeable is the difficulty in combining the advances in many domains (IoT, big data, cloud computing, machine learning), in order to provide the services needed in a smart city at reasonable parameters [15].

The remainder of this paper is structured as follows: the next section provides a literature review for machine learning techniques, clusters and cloud platforms and big data challenges, all in the context of a discussion of smart cities. Then, we propose a city scale architecture for smart cities’ needs based on Hadoop and its components. The final section presents the results obtained when testing the proposed architecture on several data sets, and some discussions, taking into account the processing speed, code reusability, scalability and fault tolerance.

## 2. Related Work

Using machine learning algorithms adds value to data supplied by sensors and meters. Smart Buildings and IoT facilitate monitoring energy consumption and environmental parameters in buildings [16]. The data is converted into information, information into knowledge, and knowledge into intelligent decisions. Examples include analysis of electricity consumption, forecasting growth areas, criminological analysis, equipment status, drawing automatically the best route for public transport users, personalized portals for citizen applications to locate various objectives (e.g., parks, ATM), etc. Also, solutions for business analysis use data from different departments that have the potential to identify new perspectives and unique solutions in service delivery, as discussed in [17], where the authors propose a general framework for the creation of e-Business models in eGovernment. 

Buildings use about 40% of the total energy, as about 75% of them are energy inefficient [18]. Using renewable energy, especially when produced on the building premises is one of the critical components of energy efficient buildings according to the EN 15232 standard [19]. There is a growing interest in decentralized energy production in urban environments, as people are discovering the potential of solar production on roofs, but also on building facades [20,21,22]. People who install their own solar power generators can act as both a user and a provider. The role of the citizen requires bidirectional communication between stakeholders and an awareness of the conditions which must be mutually respected. If building level renewable energy production is accompanied by larger photovoltaic power stations and wind power plants, this can have a positive impact on the economic and social environment [23] (e.g., reduced pollution, reduced electricity price, creation of more jobs). Some researchers argue that renewable energy can cap economic growth due to the need of backup capacity from controllable fossil generation to offset the absence of instantaneous renewable resources such as sunlight or wind [24]. But, generating more accurate power predictions is one of the fundamental research areas in renewable energies, because it can significantly reduce the costs of imbalances and the need for tertiary power reserves [25,26].

A holistic review of IoT applications has been presented in [27], including architectures (the classic three-layer architecture and the SoA-based four-layer architecture), technologies, security and privacy issues, and the integration of fog/edge computing and IoT to support the need of the applications.

CIDAP is a big data-based platform that tracks data collected from smart cities through an IoT middleware. The primary concern is the storage and processing of a significant amount of data in real time. It includes a big data repository for primary data storage, which is implemented in CouchDB, a non-relational database that uses JSON type format. Data processing is partially provided by CouchDB, but in order to process unstructured data for analysis purposes, the architecture includes Apache Spark. The platform has already been tested in SmartSantander, one of the most extensive running smart city test beds in the world [28,29,30]. 

Partly based on SmartSantander, the Organicity project proposes an Experimentation-as-a-Service (EaaS) solution, aiming to federate existing smart city platforms with a focus on crowd-sensing. The intention of the project is, that with the help of the platform, citizens will implicate themselves in identify challenges, create experiments, and find potential solutions for these problems [31]. Also, the different stake-holders can make different contributions (e.g., characterizing smart city data).

OpenIoT [32] is an open source middleware for IoT application development. It provides an API to connect to the wireless sensor network, and uses big data tools for data storage and processing. The OpenIot architecture has three plans: the physical plan—the interface with physical devices for configuring and collecting data, the virtualized plan—dealing with data storage, scheduling services and service management, and the Utility App plan—the user interface of the platform, development and configuration tools. The platform does not provide tools for real-time streaming data processing.

BASIS [33] is a big data architecture for smart cities. The architecture pays attention to multiple abstraction layers, from the most conceptual to the most technological. It uses the Hadoop mechanism to analyze data from various data sources, like file uploads and social network APIs. It uses the Hadoop file storage system along with HIVE tables and non-relational storage to store different types of data. The architecture has been tested on various use cases.

Sentilo [34] is a smart city platform that was designed for the city of Barcelona, but it has been released as open source and can be used by any city or organization. Its primary objective was to share information between heterogeneous systems and easily integrate legacy applications. The architecture manages sensors and actuators using IoT, and it uses large data tools to collect and store data. It offers a high level of scalability and interoperability. 

Using Hadoop as part of a smart city architecture attracted attention in different scientific works. A four-tier architecture that can be used for remote smart city and urban planning IoT Big Data analytics is proposed in [35]. Another architecture for smart cities that uses Hadoop and Spark open-source solutions has been proposed in [36]. The architecture is structured in three layers: the first layer collects and cleans data, the second processes data, and the third offers tools for developing user applications for the visualization of results and generating reports.

As most of the mentioned platforms do not provide real-time data streaming analysis, an exciting contribution is the infrastructure that is described in [37]. The architecture was developed for real-time processing of data provided by vehicles in traffic, and for sending recommendations to drivers, using a simulator to test the scalability and response speed of the solution. The cost element has played an essential role in the technology selection: Apache Kafka [38], being the distributed streaming solution, was selected and tested in several configurations. 

A very interesting open source platform that uses both distributed (Open Traffic Reporter, Open Traffic Analyst User Interface) and centralized services (Open Traffic Basemap Producer, Open Traffic Datastore, routing engine) is Open Traffic v2 (OTv2), that is available on github [39]. It generates OSMLR tile sets that are freely available on Amazon Web Services [40]. The platform was used as part of different grants (e.g., World Bank’s Big Data Challenge Innovation Grant) to improve traffic management it cities like Cebu, Manila or Jakarta [41].

Implementing distributed solutions (e.g., Hadoop) can be realized with the help of a Cloud platform because it reduces upfront costs, and offers both easy scaling and a content delivery network. It can be used in the development and testing phases, and even in production, as the sole solution, or as a hybrid that combines data from the customer’s premise and the Cloud. Data can be stored separately, in object-based (e.g., Amazon S3) or block-based storage (e.g., Amazon EBS) services, depending on the application.

Scaling in a cluster involves dividing the workload between the different nodes. There are also alternative approaches that replicate the same request multiple times, to be processed in parallel by the different nodes, and to accept the result from the first node in order to finish [42]. 

Cost optimization in Cloud platforms is discussed in [43]. The authors take into consideration different variables, such as virtual machine types, limits imposed by providers, and different price schemes. Considering all of these, they search for the optimal solution and also, for a quick, but adequate, approximated solution. 

In Europe, a cooperation between the European Commission and private partners has yielded an open standard platform, FIWARE. This is the standard offer for Smart City projects supported by the European Commission, as previously developed projects used proprietary or vertical solutions that were difficult to spread and applied globally. From a technical point of view, FIWARE provides “OpenStack-based Cloud capabilities and a set of tools and libraries known as Generic Enablers (GEs) with public and open-source specifications and interfaces” [44]. The platform uses a Context Broker (REST implementations) to manipulate data from various sources in smart cities. Data access is accomplished through a single API (NGSI), using the set of GE’s mentioned above. Connection to provided cloud facilities can also be achieved via a Web portal or through the command line. It is a significant investment in Europe, involving 52 partners in 13 countries with over 100 million of euros spent in 2011–2016. It continues to develop within the HORIZON2020 program. Fifteen European cities have provided real data and are connected to Fireware Lab experiments and Fireware LAB Cloud, offering free computing capacity by 16 nodes distributed in Europe. Some voices criticized FIWARE for being ”over-engineered and unnecessarily complex” [45]. The authors are advocating the necessity of using a mobile cloud system because a large part of the services in Smart City can be viewed as on-demand Internet-of-Things systems. In general, Smart Cities are facing a heavy workload; it is therefore essential to support data collection and dynamic, end-to-end resource provisioning to by IoT systems. We propose an information-centric design-based architecture that aims to deploy a publish-subscribe messaging system as a cloudlet. Smart Cities take advantage of cloud services, including public, private and hybrid solutions. Examples include the IBM SmartCloud Dubuque [46] project; it represents an intelligent solution based on Cloud, that allows citizens and companies to monitor water and energy consumption. In this case, the cloud solution enables more efficient integration and coordination of various applications on a common platform. Small towns can also benefit from the functionality offered by Cloud solution, just like the major cities.

Many smart city initiatives and frameworks have been developed as part of research projects. One of the problems with such initiatives are that, after the funding is exhausted (FP7, H2020 etc.), numerous projects do not seem to develop further on their own, and many websites are down or not updated for years so information is not as complete as it could have been (e.g., http://www.semsorgrid4env.eu, http://www.fi-ppp-outsmart.eu/, and http://www.opencities.net/content/project). Nonetheless, there are lessons to be learned from every project, especially from the articles written as part of these projects regarding the chosen technological stack and the challenges of smart-city platforms. 

A unified city scale ICT architecture is required to benefit from the full potential of a smart city [47,48,49]. In this article, in order to build a robust ICT architecture, we investigated many solutions that address smart city problems: the need for a powerful communication model, the integration and real-time processing of various fast-moving streams of data, the need to process semi-structured and non-structured data, the interoperability of different systems, and reliable scaling as processing needs increase. 

## 3. System Architecture and Components

An IoT enabled smart city architecture has three main tiers: the back-end tier (data storage and processing), the IoT peripheral nodes tier (sensors, actuators, and other embedded systems) and the middle-tier (the gateways).

For a city scale architecture to provide the space to store the data and the processing power to analyze it, a back-end architecture constructed around a relational database would show its limits rather fast. When such an architecture reaches its limits and vertical scaling does not fulfil requirements, developers resort to workarounds like denormalization, using materialized views, using partitions, or building additional caching layers on top of the database. Such solutions have the tendency to become too complicated to maintain. 

Our primary objective is to propose an architecture that is easy to maintain and is able to manage big data produced in a smart city environment, an architecture that analyses historical data, near-time data, but also real-time data. As part of this objective, we wanted to test the way Hadoop handles scalability. If scaling is linear, a smart city could start from a three-node cluster and scale when needed to thousands of nodes and get a proportional processing boost. To choose from the plethora of solutions which are potentially useful in a smart city environment and propose the architecture, we used the datasets described in Section 4 and the criteria described in Section 5 to evaluate:

- Two bulk data loading solutions: Apache Sqoop [50] vs. Oracle Loader for Hadoop [51];
- Two streaming solutions: Spark Streaming [52] vs. Apache Storm [53];
- Two NoSQL databases relevant for a smart city architecture: HBase [54] vs. Cassandra [55];
- Two NoSQL databases using two SQL query engines: Apache Phoenix [56] vs. Presto [57];
- Three Hive [58] execution engines: MapReduce vs. Tez vs. Spark [59].

**Figure 1:**
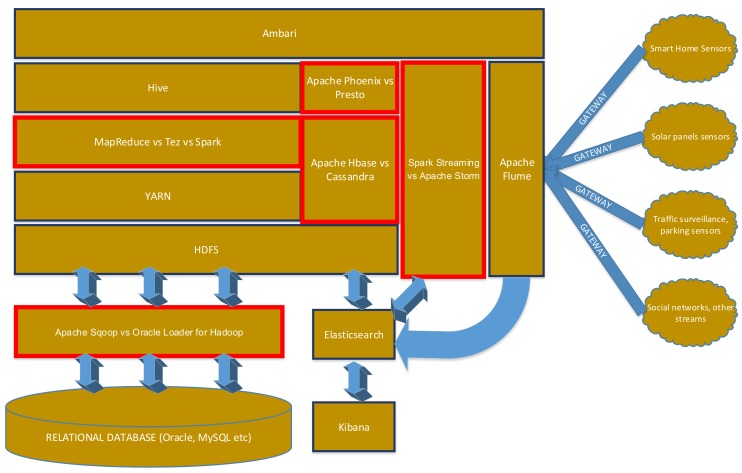
Hadoop architecture for smart cities.

The architecture is shown in Figure 1. The elements that are evaluated in this paper are highlighted in red.

In a smart city environment, most data are produced by IoT nodes which are typically resource-constrained devices that cannot exclusively rely on their own limited resources to satisfy their computing needs. Nonetheless, as the nodes, the controllers, or more commonly the gateways, become more and more intelligent, collecting, transforming, summarizing data and decision making regarding data routing and data prioritizing can be brought towards the edge, nearer to the end-user, paving the way for a fog architecture to develop alongside cloud computing [60]. There are several IoT data collection solutions [61]. For example, Stack4Things [62] proposes a data collection and inference architecture constructed using the free, open-source OpenStack cloud platform. In this approach the board (node level) is like a cloud machine instance, a compute node is like any standard or IoT enabled machine, and the controller hosts a Ceilometer collector. The board of the IoT nodes (e.g., Arduino YUN) runs a Python implemented probe that loads a monitoring plugin that talks with the Ceilometer agent of the compute node using an AMQP (Advanced Message Queuing Protocol) queue. The same communication approach is employed between the Ceilometer agent and the Ceilometer collector. The Ceilometer which is traditionally used in OpenStack deployments for customer billing, resource tracking, and alarming capabilities [63] is extended at the compute-node side using a pollster that extracts messages from the queues and at the controller-side with a dispatcher that analyses the measurements coming from the probes and which were encoded by the agent. In the Stack4Things architecture, the measurements are sent to MongoDB and the standard OpenStack Dashboard, Horizon, extended with an IoT-enabled panel, is used for data visualization. Another approach would be to send the measurements to Flume, and from there to Elasticsearch and Kibana, for indexing and visualization.

### 3.1. Resource Negotiator

YARN [5] provides resource management, security, and different governance tools, making it possible for other developers to construct data access software that runs in the cluster. Multiple projects and frameworks are taking advantage of the open source platform, being developed as part of the ecosystem to address storage, processing and scheduling needs generated by high-variety, high-volume, and high-velocity data. Even if some projects and solutions are popular, alternatives can out-perform them in various scenarios. For example, Apache Mesos [64] can be an alternative to YARN, the default Hadoop resource negotiator, when scalability is salient or when controlling not merely Hadoop jobs but a whole data center is necessary. There are incubating projects, e.g., Apache Myriad that “enables the co-existence of Apache Hadoop and Apache Mesos on the same physical infrastructure. By running Hadoop YARN as a Mesos framework, YARN applications and Mesos frameworks can run side-by-side, dynamically sharing cluster resources” [65]. Apache Spark has its cluster manager, but it can also use Hadoop’s manager, YARN, or other managers, such as Mesos.

### 3.2. Relational Stores and Bulk Data Transfers

Apache Sqoop [50] can move, using mappers, large volumes of data between relational sources like existing databases and a Hadoop cluster. Using --check-column and --last-value parameters the database and the cluster can be kept in sync. Sqoop can be started from the command line, from a script in crontab or from Oozie. Apache Flume and Apache Kafka [66] work with data from fast-moving streams generated by social media, different sensors, or as a result of reading application or server logs. Data can be delivered to various targets (HDFS, No-SQL databases, Flume) in parallel. These tools can be used to load data in HDFS, and in conjunction with Hive’s schema-on-read approach. Flume uses multiple sources and sinks to load data into the targets. These can work in parallel. The source receives an event from the exterior and stores it in a passive store, called a channel. By using this buffer, the sink, and the source work asynchronously. The sink consumes the data from the channel and loads it into the target. This approach is useful for integrating the infrastructure-based services.

### 3.3. Sensors Data Ingestion and Streaming 

Service delivery for smart cities requires applications to handle the massive volume of streaming data provided by sensors. When real-time processing with latencies in milliseconds is required, Apache Storm [53] or Spark Streaming can be used. These can be useful for processing data coming from sensors, and integrate well with a distributed message system such as Apache Kafka, that can work with hundreds of megabytes per second, from multiple clients. Similarly, Spark Streaming takes Spark beyond batch processing, as it can be used to ingest and process streams of data in real time.

### 3.4. Large-Scale Data Processing

For data processing and analytics, Apache Spark uses in-memory processing at distributed scale and can be an alternative or compliment to a Hadoop cluster, leveraging HDFS. When running in a cluster, the Spark driver runs on the master node and communicates with the cluster manager to distribute processing to the worker nodes. The manager handles the eventual failures and, after the processes are over, collects the results and passes them to the driver. As shown in [67], the primary abstraction in Spark is an RDD (Resilient Distributed Dataset), which is a collection of elements partitioned across the nodes of the cluster that can be processed in parallel using a series of operations (transformations or actions). Spark uses wide dependencies to break execution into stages, with shuffle operations between them, and narrow dependencies to create pipelined tasks. Apache Spark uses a Directed Acyclic Graph (DAG) scheduler. Consequently, the execution style is acyclic; once a stage is executed, the execution doesn’t return to that stage. Spark, by default, caches the intermediate data between stages in memory. By request, Spark can also cache an RDD inside a stage. By using RDD, Spark achieves fault tolerance by keeping track of all changes that happen on the dataset, and produces speed improvements compared to Hadoop MapReduce for algorithms that reuse intermediate results across multiple computations. Spark 2.0 extends RDD to table-equivalent DataFrame objects that can, among other things, contain row objects of structured data on which SQL queries may be run. With this approach, we can have a schema associated with the data. Thus, data can be more efficiently transported through the cluster, and queries can be more optimized. Additionally, Spark has a library for machine learning (MLlib), a library for graph-oriented analyses (GraphX), and an SQL implementation (Spark SQL) for querying structured data, offering APIs for modern programming languages (e.g., Python, Scala, Java). It can be suitable for implementing iterative algorithms, machine learning algorithms like graph processing, page ranking, logistic regression, Artificial Neural Networks (ANNs) or Bayesian Network Classifiers (BNCs), as tested in [68]. As shown by its original developers [69], besides iterative algorithms, RDD and Spark’s other significant strength is in interactive data mining. 

### 3.5. Data Storage for OLAP and OLTP

Hive is a data warehouse project that runs on top of Hadoop. It implements a query language, HiveQL, that integrates most of the SQL-92 standard, but compared to classical relational databases, can use a schema-on-read approach. This method enables data to be ingested and stored first, and then, if necessary, a table can be created over the stored data for querying purposes. The data is stored in text files, RCFiles, or in a database (by default, Apache HBase). Implicitly, the metadata is stored in Apache Derby, but other relational databases (e.g., MySQL) can be used. Hive can use Spark [70], as well as MapReduce or Tez, as its execution engine [71]. 

HBase is a NoSQL database modelled after Google’s Bigtable [72]. It offers real-time read/write access to Big Data, guaranteeing partition tolerance and consistency. The hbase shell can seem difficult to use at first. It doesn’t support SQL but uses commands such as *describe, scan*, *count or get*. To overcome this problem, distributed SQL style query engines were built. Phoenix and Presto are distributed SQL query engines that can combine data from different types of data sources in a single query. They offer RDBMS style semantics (SQL), and support transactions and user-defined functions (UDFs) based on Java Archives (JARs). Apache Phoenix is built primarily for HBase but can also access other sources such as Hive or Pig. Apache Presto is similar but can access more data sources, including Accumulo, Cassandra, Hive, MongoDB, MySQL or PostgreSQL [57]. The Phoenix client communicates directly with the HBase API, with the help of the Phoenix Co-Processor (that installs on the server side), and Zookeeper, to keep track of the available HBase region servers. 

For developers, in addition to using dedicated SQL engines, accessing data from different sources can be accomplished using different languages (Java, Scala, Python, Pig). Both Phoenix and Presto enable running SQL commands from the command line, but also from GUI clients or other products that support JDBC drivers (Apache Pig, Flume, Sqoop, Hive, and SparkSQL). In the following example we use a PIG script to access data stored in a Phoenix backed HBase table:

REGISTER /usr/hdp/current/phoenix-client/phoenix-client.jar

A = LOAD ‘default.sentiment_ratings’ USING org.apache.hive.hcatalog.pig.HCatLoader();

STORE A into ‘hbase://ratings’ using org.apache.phoenix.pig.PhoenixHBaseStorage(‘master_node_ip’,‘-batchSize 1000’).

Elasticsearch is a database engine that gained a lot of popularity lately [73]. It is an open-source, scalable, distributed RESTful search and analytics engine [74] based on Apache Lucerne, a Java written text search engine. It provides accurate results in near real-time [75] on different types of searches (single and multi-field, proximity, autocomplete, etc.), on different types of data (structured, semi-structured and unstructured). It can be extended using plugins bundled into an X-Pack [76] to support extra features (security, machine learning, reporting, etc.). Elasticsearch, with the help of the visualization tool Kibana [77], can help monitor the entire infrastructure and can also index, filter, sort, aggregate or correlate data coming from the sensors. Elasticsearch can use HDFS for long-time archival and can easily move data to and from Hadoop (including Spark, Spark Streaming, and SparkSQL) with the aid of the ES-Hadoop connector [78].

## 4. Data and Methods

The queries and the test results are in the supplementary excel file. We tested the proposed architecture on clusters built on Amazon Elastic MapReduce (EMR) with the datasets initially stored in S3 buckets. When needed, the datasets were copied from S3 into the HDFS. We started with a three-node cluster and scaled up to near real-time to evaluate the improvements. When clusters are smaller, the results are less dependent on the data distribution and access paths and more on the evaluated products. While benchmarking different products or engines to maintain similar testing environments, if the product provided a Thrift JDBC/ODBC server, the statements were run over JDBC from Java or Scala classes. Where applicable, JMeter or JProfiler were used. 

In developing the architecture, we were inspired by the “urban smartness” approach suggested in [79]. It represents a set of processes, features and technologies that are required to make a “Smart City”. They are inspired by the protocols for the evaluation the smartness of cities at European Union level. 

The smartness of the infrastructure is a crucial element in obtaining a smart city [80]. A city can optimize resources, monitor public security, and provide proper maintenance only if its infrastructure is connected and integrated: streets, roads, boulevards, parking spaces, rail lines, underground tunnels, communication lines, public lighting, traffic lights, public transportation, video surveillance systems, pipes, power lines, significant buildings, parks and recreational areas. Social media and forum data can be used to extract and synthesize tourist and citizens’ problems and experiences. It can be an efficient way to identify trending problems, but also to assess damage during emergencies [81,82,83]. By implementing the Big Data oriented solution that we propose, a city can obtain benefits by having a scalable state-of-the-art architecture that is fit to support the data and service integration of the different components of its infrastructure.

**Figure 2:**
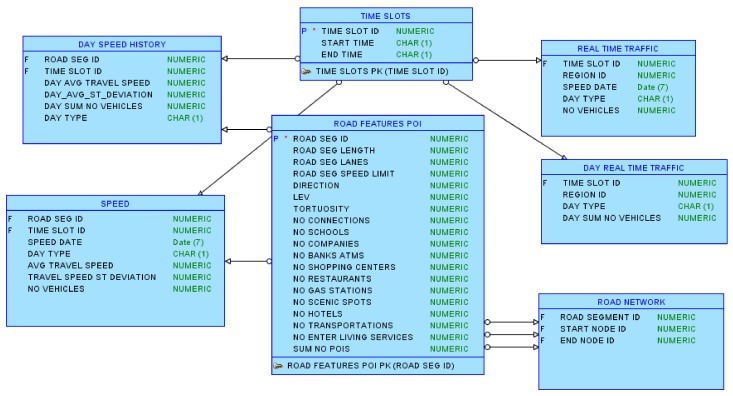
The structure of the first data set.

To benchmark the architecture components, we used four datasets that we considered a good sample of smart-cities related data. The first is comprised of data from Beijing that was extracted from GPS trajectories of taxicabs, road networks, POIs of Beijing, and video clips recording real traffic on road [84,85]. The dataset size is 866 MB and can be downloaded from [86]. In the original dataset, the data from different days was stored in different files, and the data that contained calculations was stored separately for weekdays and holidays. For simple analyses, when we loaded the dataset into the NoSQL databases, we added a column with the date to the *real_time_traffic* and *speed* tables, and a column that indicates if the day is a holiday or not to the *real_time_traffic*, *speed*, *day_speed_history* and *day_real_time_traffic* tables. We also constructed a table, *time_slots* that shows the *start time* and *end time* of each time slot (e.g., slot 0 for interval 00:00:00 00:09:59; slot 1 for interval 00:10:00 00:19:59). The structure of the database that is not entirely normalized is shown in Figure 2. This dataset was used to benchmark the OLTP elements (NoSQL databases and the query engines) for operations that require joining data and data aggregation (e.g., inner/outer joins combined sum, avg, stdev).

The second dataset was derived from a 2017 Reddit comment dataset [87] and can be found in this article’s complementary materials. The whole Reddit dataset that starts from 2005 is extensive (over 900 GB), making it an efficient solution for evaluating distributed storing and processing solutions. The 2017 subset that we used had 7.7 GB in bz2 format, or over 41 GB when uncompressed. We processed it (in Python) using the VADER Sentiment Lexicon [88] to rank every post as positive, negative or neutral. Lexicons have improved over time; the VADER lexicon was developed with the help of 10 trained people that ranked 9000 token features on a scale from “[−4] Extremely Negative” to “[4] Extremely Positive”, with allowance for “[0] Neutral (or Neither, N/A)”. It takes into consideration punctuation, capitalization, emoticons, acronyms and slang with sentimental value. For our case study, we added the scores of each token, adjusted according to the rules to the [−1, 1] interval, and calculated the compound score of over 54.3 million posts and categorized them as positive (compound score ≥ 0.5), negative (compound score ≤ −0.5) or neutral −0.5 < compound score < 0.5):

- Number of positive posts: 4,575,572, of which 17,829 were very positive (score > 0.95);
- Number of negative posts: 2,184,071, of which 8920 were very negative (score < −0.95);
- Number of neutral posts: 47,538,380.

When doing sentiment analyses, any lexicon will generate errors (false positives, false negatives) because it has limited possibilities to detect the context, including double-entendre, irony and sarcasm. For example, a sentence like “I hate you” that receive by applying the lexicon a strong negative score (−0.6114) can have a positive meaning in a particular context. 

The resulting file had 1.1 GB, 4 columns (*roundedFinalScore*, *maxPosScore*, *maxNegScore*, *postId*) and 54.3 million lines. Because it is a dataset that resembles various smart-cities use cases (including sensor outputs) that involve lots of rows and few columns, we used this dataset to test all the products using aggregate intensive operation. 

The third dataset is also included in the complementary materials and it is comprised of 5,144,791 XML documents (1.1 GB in total) that show the transactions from an online shop (the names and the emails of the customers have been anonymized) having the schema:

<xs:schema attributeFormDefault=“unqualified” elementFormDefault=“qualified” xmlns:xs=“http://www.w3.org/2001/XMLSchema”>

 <xs:element name=“record”>

  <xs:complexType>

   <xs:sequence>

    <xs:element type=“xs:string” name=“Customer”/>

    <xs:element type=“xs:string” name=“Email”/>

    <xs:element type=“xs:string” name=“PurchaseDate”/>

    <xs:element type=“xs:float” name=“Price”/>

    <xs:element type=“xs:string” name=“ProductIDs”/>

   </xs:sequence>

  </xs:complexType>

 </xs:element>

</xs:schema>

The dataset was used to test the XML processing capabilities of the OLAP solution (e.g., *split*, *lateral view*, *explode*).

To evaluate the streaming solutions, we used the second dataset mentioned above in addition to a fourth dataset: weather data captured in real time from [89], using a developer key and a Python program (columns: year, month, day, precipitations, maxTemp, meanTemp, minTemp). The data was sent line by line from ten sources to Apache Flume (e.g., *python getweather.py | nc <ip_master_node> <netcat-collect.port>*), to be consumed by Spark Streaming or Apache Storm. A small sample is included in the complementary materials.

## 5. Results and Evaluation

When comparing two or more real-time or micro-batching Big Data products, speed is, of course, important. But even more important is the way the speed scales when new nodes are added to the cluster. Even if one product is faster on the initial cluster, another might out-perform it when more nodes are added. Other important things to consider are support, innovations, features, or the way a product integrates into the solution. Underpinned by our Big Data projects experience, to compare the products mentioned above we propose the following criteria based framework:

1. Speed increments when scaling to a cluster with more nodes (50%)
2. Processing speed over the datasets (15%)
3. The reusability of the code between the components of the architecture (15%)
4. Innovations in the past 36 months (e.g., DataFrames in Spark) or important extra features (10%)
5. Releases in the past 12 months (5%)
6. Support/interest shown in online communities (5%)

For the first criterion, the scores are proportional to 100. A maximum score would indicate a perfect linear scalability when switching from a three-node cluster to a five-node cluster (e.g., the query time halves when the working nodes double). For the second criterion, the fastest product receives a score of 100 and the rest, proportional to 100, according to the average speed difference calculated for each test executed on the three-node cluster (with default settings). For the third measure, scores can be 100 (very high reusability), 75 (high reusability), 50 (some reusability), 10 (no reusability). For the fourth, if at least one innovation was identified, the product receives 75 points; if it has an important extra feature compared to the other analyzed products it receives 25 points. For the fifth criterion, if a product had at least two releases in the past 12 months, it receives 100 points; if there was one release it receives 75 points. An active and vibrant online community can show interest in a product and can help drive it forward, so for the sixth measure, if there is an active official support forum the product receives 50 points. The remaining 50 points are awarded proportionally to the product that has had the most answered questions on StackOverflow in the past 12 months. The final composed score of each product is calculated according to the weighting system in the brackets. There are also other criteria that are taken into consideration by the team when choosing a solution, but some are harder to be quantified in a model, e.g., security features or previous experience with a product compared to another.

The scores for the analyzed products are shown in Table 1 and Table 2.

The advantage of these measures is that they can be easily used to compare other products that offer similar features. For this smart city and IoT oriented use case, for NoSQL databases, we looked into HBase and Cassandra, but for other situations, for example, a business-oriented use case, we could evaluate CouchDB and MongoDB.

When testing on Amazon Elastic MapReduce (EMR), we used the following configurations:

- 1 node pseudo cluster—8 vCPU, 15 GiB memory, 80 SSD GB storage;
- 3 node cluster: 1 master—8 vCPU, 15 GiB memory, 80 SSD GB storage EBS Storage, 2 cores: 8 vCPU, 15 GiB memory, 80 SSD GB storage;
- 5 node cluster: 1 master—8 vCPU, 15 GiB memory, 80 SSD GB storage EBS Storage, 4 cores: 8 vCPU, 15 GiB memory, 80 SSD GB storage.

When testing scaling, we didn’t use cluster resize for data balance concerns. The three-node cluster and the five-node cluster were started from scratch, and the data was loaded in HDFS each time. 

We choose Hive as the data warehouse solution with Apache Tez as its main execution engine, because in outperformed the other solutions (Table 3, Table 4, Table 5 and Table 6). Apache Spark as the execution engine is also a good option; it has scaling potential because executing Hive is executing MapReduce primitives on Spark improving the queries that involve multiple reducer stages, similar to Tez [59]. Classical MapReduce is not a good solution; it was outperformed, and it is considered deprecated in the newest Hive versions and Tez as a backup. With this approach, for data processing, Spark, compared to legacy Hadoop MapReduce, solves inefficiency in areas such as iterative machine learning and interactive data mining by not HDFS storing intermediate stages. Using Hive with Spark can help in standardizing the execution backend, especially when Spark is also used for other processes that run in the cluster, improving operational management and maintenance, making debugging easier. Furthermore, Hive exposes a thrift server and JDBC/ODBC driver so it can be accessed like any other database server. Nevertheless, even with Spark or Tez as execution engines and with the introduction of transactions with ACID semantics (starting from version 0.14), Hive should not be used when a low latency OLTP database server is needed. A select with “group by” over a 1.1 GB file takes 100 s (the first run) on a three-node cluster and 85 on a five-node cluster. 

For real-time processing, we included Apache HBase in the architecture, a NoSQL database that emphases consistency and partition-tolerance and natively works with HDFS. For comparison, Cassandra uses its own filesystem, Cassandra File System (CFS), so if we needed to copy data from HDFS to CFS, we would need to use a tool like DSE. As shown in Table 7, on the datasets, HBase used with Phoenix proved to be slower compared to Cassandra, but demonstrated better scaling capabilities. HBase implements a four-dimensional data model that uses the following components: row key, column family column qualifiers and data value version. The dataset that we used and the more analytical oriented queries seem to favor Cassandra, so Cassandra and Presto outperformed HBase and Phoenix, but the latter two showed a better scalability potential. Also, Presto has the advantage that it can connect to more data sources (Hive, Cassandra, MongoDB, MySQL etc.) and can join tables from multiple sources.

For streaming, even though Storm provided slightly better latencies during tests, we chose Spark Streaming for standardization reasons, as Spark was included in the architecture and the same code can be used for both batch processing and stream/micro-batch processing. Starting with version 2.1, Spark also offers Structured Streaming, “fast, scalable, fault-tolerant, end-to-end exactly-once stream processing without the user having to reason about streaming” [90]. Both Spark Streaming and Storm provide a similar fault tolerance. 

For importing/exporting relational data into/from HDFS, we tested Sqoop and Oracle Loader for Hadoop (OLH)*.* The functionalities of the two products are similar, with OLH proving better support for the Oracle Databases. We chose Sqoop for the architecture because it is more general compared to OLH (since it can be used with other data sources besides Oracle). 

We also included Ambari in the architecture because it provides a smooth, interactive way to monitor and administer the cluster, to start or stop components, and to change the memory allocated for a node or a container or setup alert thresholds. We selected Ambari over Hue because it provides more features and a better user interface.

## 6. Discussions and Further Research

This article proposes a robust architecture for managing a Smart City that can seamlessly handle a heterogeneous field of complex applications that need to interact, process various fast-moving heterogeneous streams of semi-structured and not-structured data, and provide reliable and cost-effective scaling as processing needs increase, gaining the ability to handle limitless concurrent tasks. The need of such an architecture is discussed in the literature [47,48,49]. Some papers, even if they acknowledge the importance of distributed processing only mention a cluster or a NoSQL database as part of the architecture [13,91], without drilling down.

The Big Data Hadoop centered environment changes very quickly, and some research can be obsolete in 2–3 years. For example, in a 2015 article, it was stated that NoSQL systems “support only simple query interfaces, i.e., key-based access or single-table queries requiring that the application developers have to implement more complex operations such as joins themselves” [92]. This is no longer true; as discussed in this paper, SQL-type queries can be implemented in NoSQL databases, sometimes with the help of query engines that can combine, using a single query, data from multiple sources, joining a table from a NoSQL database with another one from a different NoSQL database or even from a distributed data warehouse solution such as Hive. Query engines like Phoenix can also be used to enable ACID (Atomicity, Consistency, Isolation, Durability) compliant tables over NoSQL tables (*set phoenix.transactions.enabled* = *true*). With Hive, on the other hand, the data warehouse solution can natively provide full ACID semantics at the row level [93]. Nonetheless, such distributed solutions are not even mentioned in recent works where the authors test transactional services in NoSQL databases, propose their own middleware layer over Riak [94], and test the solution using data from the Council of London for public transportation of bus services [95]. 

We discussed all the main architectural needs: bulk data loading, data ingestion, data streaming, OLTP and OLAP. For the architecture, we only considered components that are open-source and have no license costs, and analyzed their performance in different scenarios. For each component of the architecture, there can be tens of possible candidates, each with its strengths and weaknesses. Consequently, we developed the framework described in Section 5, and used it to evaluate the components described in Section 3 and in Figure 1, based upon the Smart Cities relevant datasets described in Section 4. 

The proposed architecture has taken into account the requirements for storing and processing large data volumes associated with a smart city. Benchmarking any product, including desktop computers, servers, data center architectures and mobile devices, always has its biases, fallacies and pitfalls [96], partly because it is hard to classify and match real-life scenarios. This can mislead architects to identify the wrong bottlenecks and make improper trade-offs [97]. When benchmarking distributed systems, things are even more complicated because performance is dependent on data distribution, the path to data of a given query, and the heterogeneous network that connects the nodes. 

We aim to improve the results by testing on multiple sets of complex data and by developing the framework to also take into considerations the optimization of combinations of components from different categories. Also, one of the shortcomings of the tests was that we only tested scaling from a three-node cluster to a five-node cluster. To get a better view of the scaling capabilities, tests should also be conducted on clusters with substantially more nodes.

Machine learning based programs should be developed and benchmarked because they can offer insights based on the data that is ingested by the cluster. For example, meteorological, environmental and astronomical factors could be aggregated with the actual measured power of the power panels in order to predict energy production at different lead times (usually up to 72 h). Having access to multiple readings from different PV generators could lead to improved predictions by identifying clusters of nearby generators that behave similarly. Many studies take into consideration only historical readings together with other variables from the PV generator for which they generate predictions [25,98]. Having identifying generators that behave similarly could improve predictions by adjusting the predictions using a collaborative algorithm for newly installed PV generators, for which there is limited historical data. Also, having continuous streams of weather data could help in highly focused, customized nowcasting. 

To expand the text and sentiment analyses problem, we are working on a key/value approach to construct a dictionary (stored in a NoSQL database) in which every token from the corpus received an index. The key is the position in the dictionary of the token and the value of its mean sentiment rating. For sentiment values analyses, the order of the words is essential, so we need to store the position in the text as an index. This approach makes storing text in a multi-dimensional vector space model (VSM) possible. We can store for every word, besides the average sentiment rating, the number of occurrences or the frequency-inverse document frequency (TF-IDF) that shows the importance of the word in the document. This enables running supervised or non-supervised machine learning techniques, in order to discover similar posts or connections between the posts using metrics like the Euclidean distance or the Cosine similarity.

## Figures and Tables

**Table 1:** Scores for the analyzed products (part 1)—the details regarding the tests, including the queries, are in the Excel file included in the Complementary Materials of the article. In the tab dedicated to the criteria, in the cell comments, there are additional explications regarding how the score was calculated.

Criteria	Hive with MR	Hive with Tez	Hive with Spark	Oraloader	Sqoop
**1. Speed increments when scaling to a cluster with more nodes (50%)**	60.08	74.16	67.70	0.00	0.00
**2. Processing speed over the datasets (15%)**	34.80	100.00	62.20	100.00	93.23
**3. The reusability of the code between the components of the architecture (15%)**	50.00	50.00	50.00	10.00	50.00
**4. Innovations in the past 36 months (e.g., DataFrames in Spark) or important extra features (10%)**	75.00	100.00	75.00	0.00	15.00
**5. Releases in the past 12 months (5%)**	100.00	100.00	100.00	75.00	75.00
**6. Support/interest shown in online communities**	100.00	100.00	100.00	50.00	100.00
**Final Score**	**60.26**	**79.58**	**68.18**	**22.75**	**31.7345**

**Table 2:** Scores for the analyzed products (part 2)—the details regarding the tests, including the queries, are in the Excel file included in the Complementary Materials of the article. In the tab dedicated to the criteria, in the cell comments, there are additional explications regarding how the score was calculated.

Criteria	Cassandra + Presto	HBase + Pheonix	Spark Streaming	Storm	Ambari	Hue
**1. Speed increments when scaling to a cluster with more nodes (50%)**	51.30	75.85	100.00	100.00	0.00	0.00
**2. Processing speed over the datasets (15%)**	100.00	34.43	99.00	100.00	0.00	0.00
**3. The reusability of the code between the components of the architecture (15%)**	75.00	75.00	75.00	50.00	0.00	0.00
**4. Innovations in the past 36 months (e.g., DataFrames in Spark) or important extra features (10%)**	0.00	0.00	100.00	0.00	15.00	0.00
**5. Releases in the past 12 months (5%)**	100.00	100.00	100.00	100.00	100.00	100.00
**6. Support/interest shown in online communities**	100.00	75.00	100.00	71.50	100.00	75.00
**Final Score**	**61.9**	**63.0895**	**96.1**	**81.075**	**11.5**	**8.75**

**Table 3:** Test results for execution time (HiveQL with MapReduce as execution engine, the queries can be found in the Complementary Materials).

Execution Time (s)	HiveQL with MapReduce as Execution Engine				
	Query 1	Query 2	Query 3	Query 4	Query 5
1 node	139.9	102.467	7150	2440	48.15
3 node cluster	80.9	100.69	6107	1401	40.7
5 node cluster	57.16	85.04	5803	1190	32.5

**Table 4:** Test results for execution time (HiveQL with Tez as execution engine, the queries can be found in the Complementary Materials).

Execution Time (s)	HiveQL with Tez as Execution Engine				
	Query 1	Query 2	Query 3	Query 4	Query 5
1 node	219	118.53	1487	1250.93	27.07
3 node cluster	51.67	79.48	780.53	708	20.1
5 node cluster	43.7	40.25	530.38	600	12.5

**Table 5:** Test results for execution time (HiveQL with Spark as execution engine, the queries can be found in the Complementary Materials).

Execution Time (s)	HiveQL with Spark as Execution Engine				
	Query 1	Query 2	Query 3	Query 4	Query 5
1 node	263	123.98	2967.8	2001	31.644
3 node cluster	80	101.21	1735.6	1380	24.3
5 node cluster	51	83.7	1250.3	1081	18.2

**Table 6:** Test results for execution time (Spark 2.1: spark-submit, Spark SQL over an HDFS stored file and over an Hive stored table, the queries can be found in the Complementary Materials).

Execution Time (s)	Spark 2.1 (Spark-Submit, Spark SQL over HDFS Stored File)			Spark 2.1 (Spark-Submit, Spark SQL over the Hive Stored Table)		
	Query 1	Query 2	Query 3,4,5	Query 1	Query 2	Query 3,4,5
1 node	13	25	n/a	12	23	n/a
3 node cluster	48.98	80	n/a	40.2	61	n/a
5 node cluster	38.3	57.9	n/a	31.7	43.5	n/a

**Table 7:** Test results for execution time (the queries can be found in the Complementary Materials).

	Hbase and Phoenix				Cassandra and Presto			
	Query 1	Query 2	Query 3	Query 4	Query 1	Query 2	Query 3	Query 4
**3 nodes processing speed (s)**	48.5	2.32	80.2	78.5	40.1	2	25	13
**5 nodes processing speed (s)**	27.9	1.79	58	50.5	34.3	1.85	20	10

## Supplementary Materials

The following are available online at http://www.mdpi.com/1424-8220/18/4/1181/s1.
